# Supplementary material for: V‐ATPase Disassembly at the Yeast Lysosome‐Like Vacuole Is a Phenotypic Driver of Lysosome Dysfunction in Replicative Aging
Source: Aging Cell. 2025 Jan 16;24(5):e14487. doi: 10.1111/acel.14487 (PMC12074022; doi:10.1111/acel.14487)
Supplement: Supplementary file 2 — Figure S2 [file ACEL-24-e14487-s001.pdf]

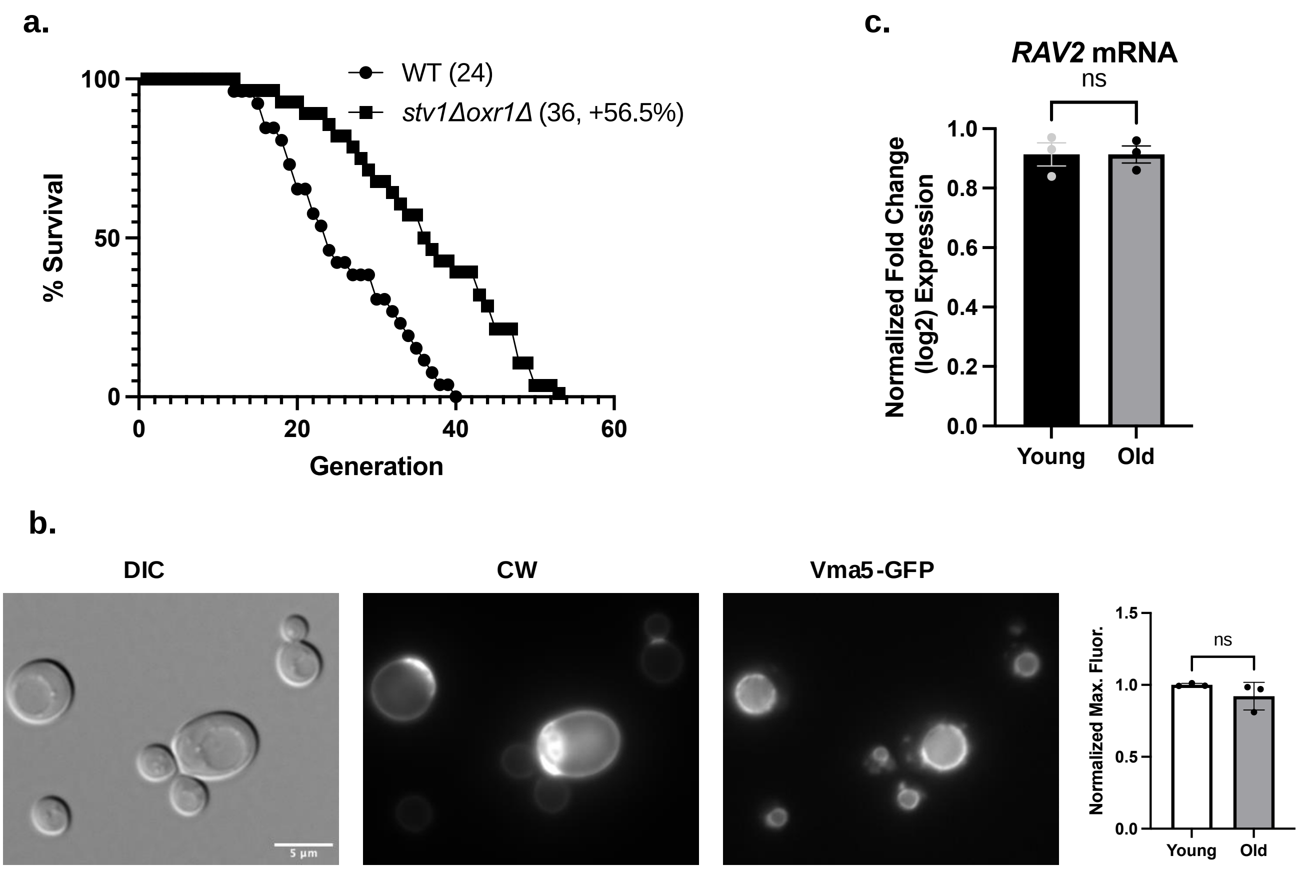

Supporting information Figure 2: a. Kaplan-Meier curves comparing the RLS of *stv1*Δ *oxr1*Δ (squares), and wild-type cells (circles). 26 wild-type cells were scored and 28 *stv1*Δ *oxr1*Δ. $p < 0.001$. b. BY4742 *stv1*Δ *oxr1*Δ cells expressing Vma5-GFP grow in SC with 2% glucose. CW was used to visualize bud scars. Normalized maximum fluorescence was obtained through line scan quantitation of Vma5-GFP using FIJI as in **Figure 1**. Means +/- s.e.m. of three biological replicates are shown; each replicate is represented by a dot. Significance was calculated by unpaired Student's *t* test and the difference was not significant (n.s.). c. Quantitative RT-PCR comparing expression of *RAV2* mRNA between young and old cells, using actin as a control. The statistical difference was not significant (n.s.) by unpaired t-test..
